# Nonsense-Mediated mRNA Decay: Pathologies and the Potential for Novel Therapeutics

**DOI:** 10.3390/cancers12030765

**Published:** 2020-03-24

**Authors:** Kamila Pawlicka, Umesh Kalathiya, Javier Alfaro

**Affiliations:** 1 Edinburgh Cancer Research Centre, University of Edinburgh, Edinburgh EH4 2XU, UK; 2 International Centre for Cancer Vaccine Science, University of Gdansk, 80-308 Gdansk, Poland; umesh.kalathiya@ug.edu.pl

**Keywords:** Nonsense-mediated mRNA decay, Premature termination codon, Cancer, Neoantigens, NMD inhibition

## Abstract

Nonsense-mediated messenger RNA (mRNA) decay (NMD) is a surveillance pathway used by cells to control the quality mRNAs and to fine-tune transcript abundance. NMD plays an important role in cell cycle regulation, cell viability, DNA damage response, while also serving as a barrier to virus infection. Disturbance of this control mechanism caused by genetic mutations or dys-regulation of the NMD pathway can lead to pathologies, including neurological disorders, immune diseases and cancers. The role of NMD in cancer development is complex, acting as both a promoter and a barrier to tumour progression. Cancer cells can exploit NMD for the downregulation of key tumour suppressor genes, or tumours adjust NMD activity to adapt to an aggressive immune microenvironment. The latter case might provide an avenue for therapeutic intervention as NMD inhibition has been shown to lead to the production of neoantigens that stimulate an immune system attack on tumours. For this reason, understanding the biology and co-option pathways of NMD is important for the development of novel therapeutic agents. Inhibitors, whose design can make use of the many structures available for NMD study, will play a crucial role in characterizing and providing diverse therapeutic options for this pathway in cancer and other diseases.

## 1. Introduction

### 1.1. The Nonsense-Mediated mRNA Decay (NMD) Pathway and Machinery

The precise regulation of genetic information, as it is passed from gene to transcript to protein, is crucial for the survival of cells and organisms. From a single gene, multiple mature messenger RNA (mRNA) transcripts arise through alternative pre-mRNA, resulting in mature species with differences in both the coding and non-coding regions [1]. Even beyond the end-points of mRNA transcription, the quality and quantity of mRNAs in cells is tightly controlled through various pathways [2]. Nonsense-mediated mRNA decay (NMD) is a critical cellular surveillance mechanism that recognizes and eliminates aberrant RNAs containing premature termination codons (PTC) or abnormally long 3′ untranslated regions (UTRs). NMD was first found to affect one-third of the mutated mRNAs [2]. Transcripts with destabilizing PTC in their coding region are products of endogenous genes with nonsense or frameshift mutations, pseudogenes [3], or from alternative splicing events leading to intron retention or inclusion of PTC-containing exons [4]. To avoid producing C-terminally truncated proteins that can have deleterious effects for the organism, those transcripts harbouring PTC are recognized and subsequently degraded [5,6]. 

In mammalian cells, the discrimination of PTC-containing transcripts depends on the position of PTC in mRNA. Transcripts containing PTC at least 50–55 nucleotides upstream of the last exon-exon junction are recognized as “premature” and degraded through NMD. As a caveat, this definition changes across the species. In *Saccharomyces cerevisiae*, PTC is defined independently of exon boundaries [5]. In another variation, the presence of introns is not necessary to define PTCs in *Drosophila* or in *Caenorhabditis elegans*, which shows a mechanistic diversity in the initiation of the NMD pathway [5].

NMD is a cytoplasmic and translation-dependent process. During pre-mRNA splicing, a multi-subunit protein complex, spanning ∼20–24 nucleotides, is deposited upstream of the exon-exon junction; the exon junction complex (EJC). Associated to mRNA, EJCs are transported into the cytoplasm, where the force of the ribosome, as it translates the transcript, is sufficient to remove the EJCs. Transcriptome-wide analysis and biological studies showed that EJCs are not loaded equally across all exon junctions of a transcript [7]. During translation of a normal transcript, the stop codon in the last exon ensures that no EJCs remain on the mRNA upon translation termination. The position of the ribosome at the end of the transcript is also important for translation termination, where interactions to proteins bound to the mRNA poly(A) tail and release factors are required. A stalled ribosome at a PTC leaves remaining downstream EJCs [2] and a distance to the 3′-end and poly(A) tail may be too large to facilitate termination. The resulting delayed release of the ribosome from the transcript affords the time needed to assemble NMD-related proteins and recruit other cofactors [8].

#### 1.1.1. The NMD Machinery

The NMD pathway was first elucidated using unbiased genetic screens from *Caenorhabditis elegans* and *Saccharomyces cerevisiae* [9,10]. Seven genes were identified in nematodes, termed *SMG1–7* (suppressor with morphological effect on genitalia proteins 1–7). Mutations to SMG were non-lethal, indicating that NMD is not essential in nematodes [9]. Three orthologous genes to *SMG2, SMG*3 and *SMG4*, *UPF1–3* (up-frameshift 1–3), were identified in *S. cerevisiae* [10]. Homology searches continued to identify orthologous genes in other species, including *Arabidopsis*, *Drosophila* and mammals **[11]**.

**Figure 1:**
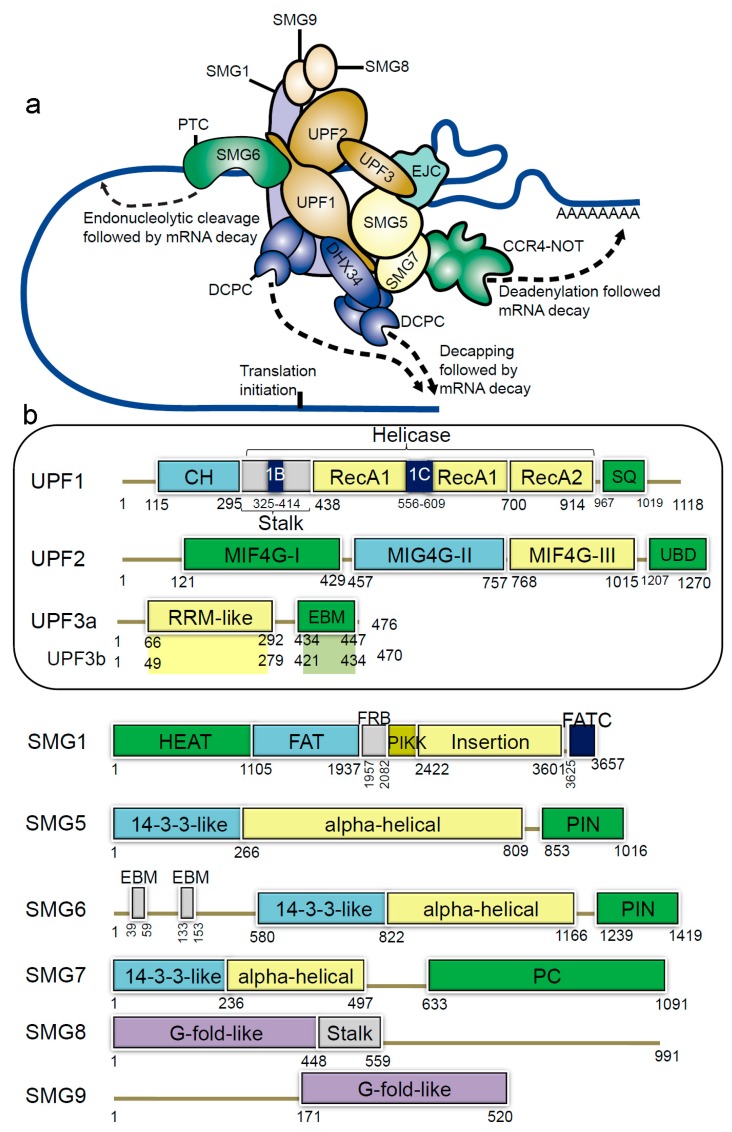
Schematic representation of domains and motifs of the nonsense-mediated mRNA decay (NMD) factors. (**a**) The NMD complex UPF: up-frameshift; SMG: suppressor of morphogenetic effect on genitalia; DHX34: DEAH box polypeptide 34; DCPC: the decapping complex; EJC: exon junction complex; CCR4-NOT: carbon catabolite repressor protein 4 (CCR4)–NOT deadenylase complex [31]. (**b**) For the UPF and SMG proteins: CH: cysteine-histidine rich domain; Stalk: RecA1 domain by two long ‘stalk’ helices; RecA1 and RecA2: RecA-like domains; 1B and 1C: subdomains within the helicase core; SQ: serine-glutamine rich domain; RRM: RNA recognition motif; EBM: exon junction binding motif; MIF4G: middle of 4G-like domains; UBD: UPF1-binding domain; PIN: PilT N-terminus domain; PC: C-terminal proline-rich region; HEAT: Huntingtin, elongation factor 3 (EF3), protein phosphatase 2A (PP2A), yeast kinase TOR1 domain; FAT: focal adhesion kinase domain; FRB: FKBP12-rapamycin-binding; PIKK: phosphatidylinositol 3-kinase-related protein kinase domain; FATC: C-terminal FAT domain; G-fold-like: domains involved in dimerization between SMG8-SMG9 [31,32,33].

In humans, NMD members include the hUPFs—human up-frameshift (UPF) proteins (UPF1, UPF2, UPF3a and UPF3b), the suppressors with morphological effects on genitalia proteins (SMG1, SMG5, SMG6, SMG7, SMG8 and SMG9), and the exon junction complex (EIF4A3, MAGOH, RBM8A and Barentsz (BTZ)) (Figure 1a) [2,12,13,14]. The EJC complex recruits the evolutionarily conserved UPF proteins and plays an essential role in NMD [15]. During the pioneer round of translation, some EJC components are displaced by the ribosome, and this positional information by EJC is preserved until the mRNA is translated [15,16]. In the presence of a PTC, translation pauses upstream of an EJC and the eukaryotic release factors (eRF) physically bind and recruit UPF1 (the RNA helicase) [17,18,19]. The eRFs recognize the stop codon, and when the mRNA stop codon enters the ribosomal A site, the termination of the protein synthesis occurs. The single eukaryotic class-I RF eRF1 recognizes all three (UAG, UGA, UAA) stop codons [20].

Initiation of the NMD pathway leads to remodelling of the surveillance complex (SURF), which includes the UPF1, SMG1, eRF1 and eRF3 proteins. UPF3b attaches to the EJC and anchors UPF2. The SURF complex binds with the UPF2, UPF3b and an EJC downstream of the PTC, forming the decay-inducing complex (DECID) [21]. Along with the UPF proteins the SURF complex promotes the phosphorylation of UPF1 by SMG1. In contrast, for the dephosphorylation of UPF1 a multiprotein complex composed of SMG5, SMG6, SMG7 and protein phosphatase 2A is required [22]. Allowing for the fine-tuning of the NMD activity, the UPF3a protein inhibits NMD, and this activity is regulated by the UPF3b protein [23].

The main component of the NMD machinery is the UPF1/SMG2 protein, an ATP-dependent RNA helicase, which undergoes cycles of phosphorylation and dephosphorylation that are essential for NMD progression. The UPF1 protein is involved in the translation termination complex, when an EJC lies downstream of a termination event. UPF1 undergoes a large conformational change upon binding with UPF2 protein, which activates its RNA-helicase activity [24,25,26]. Once the RNA-helicase is active, the RNA is exposed for degradation. The DEAH box polypeptide 34 (DHX34; Figure 1a), an RNA helicase of the DEAH box family, associates with several components of the NMD complex in cell lysates, and preferentially binds with the hypophosphorylated UPF1 [27,28,29]. It is proposed that DHX34 is involved in the activation of UPF1 phosphorylation, and mediates a change in interaction patterns within the NMD, which propagates NMD activation [28,29,30].

There are many pathways that lead to degradation of NMD-targeted RNAs. Studies show that in yeast, PTC-containing transcripts are degraded predominantly through deadenylation-independent process involving decapping by the Dcp1p/Dcp2p enzyme and 5′–3′ exonucleotic digestion by Xrn1p [6,26]. In human cells, those transcripts are degraded through multiple mechanisms, such as endonucleolytic cleavage [27], exosome mediated 3′–5′ decay **[30]** or deadenylation-dependent decapping [26]. Lykke-Andersen et al. performed a transcriptome-wide identification of NMD substrates and their 5′–3′ decay intermediates to establish that SMG6-catalyzed endonucleolysis widely initiates the degradation of human nonsense RNAs, whereas decapping is used to a lesser extent [31].

#### 1.1.2. Structural Insights of NMD Components at a Glance

**Figure 2:**
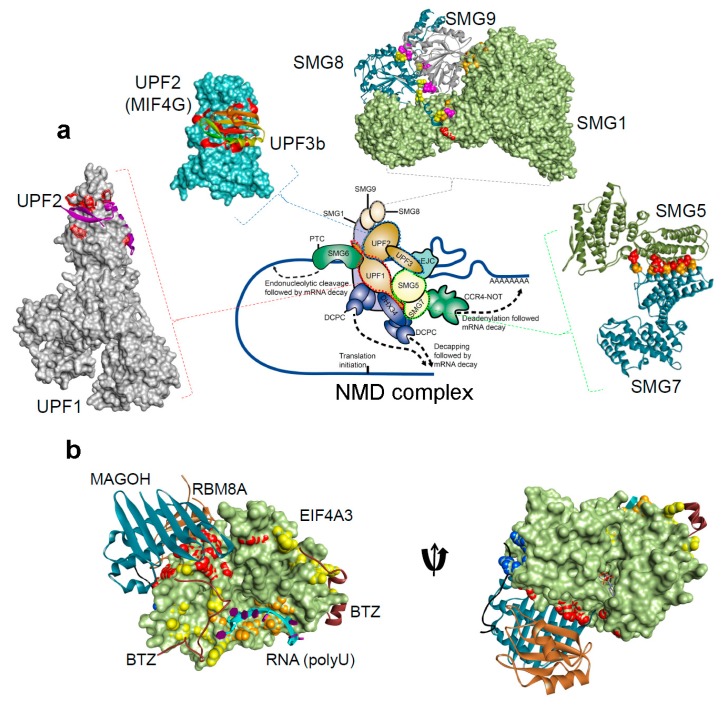
The protein-protein and protein-RNA binding interface for the NMD components, from the Protein Data Bank database (http://www.rcsb.org/pdb) [31,32,33,34,36,103,104,105,106,107,108,109,110,111,112]. (**a**) NMD pathway schematic representations, and protein-protein interactions form the complex: UPF1-UPF2 (PDB: 2wjv) [34], UPF2-UPF3b (PDB: 1uw4) [36], SMG5-SMG7 (PDB: 3zhe) [105,112], SMG8-SMG9 (PDB: 5nkk) [106], SMG1–SMG8–SMG9 (PDB: 6syt) [107]. (**b**) The exon junction complex; Mago-Y14-eIF4AIII-Barentsz-UPF3b (PDB: 2xb2) [108] (Table 1). The H-bond analysis was performed using BIOVIA Discovery Studio Visualizer program [Dassault Systemes, BIOVIA Corp., San Diego, CA, USA].

The UPF1 protein has a conserved cysteine-histidine-rich domain (CH-domain), followed by two RecA-like domains (RecA1 and RecA2; helicase region), and a SQ (serine-glutamine) domain (Figure 1b) [33,34]. From the structural analysis it is known that binding of UPF2/UPF3 protein to the CH-domain of UPF1 activates UPF1 ATPase and the helicase activities [35] (Figure 2). The UPF2 structure consists of four core regions, three domains are the middle portion of eukaryotic initiation factor 4-gamma (MIF4G-1, 2 and 3) domains and a C-terminal domain. This C-terminal domain of the UPF2 protein plays an important functional role, as it binds to the UPF1 CH-domain, enhancing its helicase activity [34]. Particularly, the MIF4G-3 domain interacts with the RRM (RNA recognition motif) domain of the UPF3b protein (Figure 1b) [36], as well as the SMG1 protein interacts with the MIF4G-3 domain at the same time as UPF3b, but in a non-competitive way [37]. Both UPF3a and UPF3b proteins do not show direct binding to the RNA, despite having a RNP domain (ribonucleoprotein or RRM) at the N-terminus (Figure 1b) [38].

The UPF1 protein is phosphorylated by the phosphoinositide 3-kinase related kinase (SMG1) [39]. SMG1 further associates with two cofactors, SMG8 and 9, and eukaryotic release factors eRF1 and 3a [21]. As a result the phosphorylation of UPF1 recruits SMG5/6/7 proteins, and these recruited components share a phosphoserine-binding domain [40]. The functional dependency between phosphorylation or dephosphorylation cycle and the ATPase or the helicase activities of the UPF1 protein, is an interesting area that needs investigation. The interactions between SMG5-SMG7 results in a stable heterodimer complex [41]. Composing of two EJC-binding motifs (EBMs) the SMG6 protein harbours an endonuclease activity that cleaves the PTC-mRNA (Figure 1b) [42]. Identifying the structural and the functional relationship between UPF1, SMG5, SMG6, SMG7 and their interacting proteins would be an interesting area to investigate [43].

#### 1.1.3. NMD Target Selection, more than just Coding Transcripts

Despite an increased understanding of the NMD process, questions remain around the rules governing the NMD target selection. Varying between the organism or cell type, ~5–20% of the transcripts can be subjected to NMD and these targets extend beyond the classical understanding of this machinery. Beyond mRNA with PTC in coding regions, a number of features can target different RNA-species for degradation by NMD. Additional targets can be classified into three main categories: (1) transcripts with destabilizing PTC arising in pseudogenes [5], or from alternative splicing events leading to the intron retention or inclusion of PTC-containing exons [4]. (2) Transcripts with limited or no clear coding potential, such as small RNAs derived from intragenic regions [44], long non-coding RNAs [31] and mRNAs of inactivated transposons [5]. (3) Transcripts with upstream open reading frames, or with abnormally long 3′UTRs, or wild-type mRNAs with no atypical features [5]. It has been demonstrated that the NMD process can occur even without the presence of EJC bound to the mRNA. In this pathway, UPF1 binds to the 3′ UTRs of the transcript and interacts with exon–exon junction components found in the cytoplasm or EJC stably associated with 3′ UTRs. The mechanism of this process is still not fully understood.

Lykke-Andersen et al. performed a transcriptome-wide identification of NMD substrates in HEK293 cell line and found that genes hosting small nucleolar RNAs (snoRNAs) and microRNAs (miRNAs) were significantly enriched among NMD substrates. The researchers hypothesized that snoRNA host genes need to be highly transcribed to regulate the high demand for snoRNA production and that the expression of individual snoRNAs and their cognate spliced RNA can be uncoupled through alternative splicing and NMD [45].

Studies of long non-coding RNAs indicate that many contain long regions located downstream of the stop codon that are unprotected by ribosomes. Considering that a long 3′ UTR triggers NMD, translation termination upstream of these ribosome-free regions gives a mechanistic explanation for the recognition and elimination of non-coding RNAs by the NMD pathway [46].

### 1.2. NMD as a Crucial Regulator of the Transcriptome 

NMD plays a role in the natural maintenance and regulation of the abundance of a large number of cellular RNAs. The pathway targets ~10% of unmutated mammalian mRNAs acting as a regulator of cellular adaptation to environmental changes, differentiation and cell survival [47]. For example, NMD plays known roles in the homeostasis of the cell, in embryonic development, in cellular response to stress and in regulating the immune response. As a consequence, the NMD response must be under a strict control in order to avoid undesirable alterations to the gene expression program of cells and tissues.

#### 1.2.1. NMD in the Maintenance and Homeostasis of the Cell 

Through evolution, some organisms have become dependent on the NMD pathway for the maintenance and homeostasis of normal cellular transcripts. This was not the case for nematodes or yeast where mutations in the main NMD components lead to discrete phenotypes [8,9]. However in humans, RNAi-based knockdowns on NMD genes showed that this pathway plays a crucial role in cell cycle regulation [48], cell viability [49] and the response to amino acid starvation [50]. Furthermore, NMD components have NMD-independent functions, such as the DNA damage response [51], maintenance of the telomere integrity [48], regulation of calcium metabolism [1], viral infection, replication [52] and tumourigenesis.

NMD also affects transcripts encoding splicing regulators, such as serine-arginine (SR) proteins. SR proteins interact with exonic enhancers stimulating exon inclusion. These factors were shown to autoregulate their own expression by creating a negative-feedback loop. For example, abnormally high expression of SC35 induces alternative splicing of its mRNA to an isoform harbouring a premature termination codon, which then gets degraded by NMD. Nonsense-mediated mRNA decay acts as a biological switch that removes alternatively spliced mRNAs when the protein product is not needed. Considering that one-third of all splice variants produced by human genes harbour PTC, the NMD process also has function in regulation of “alternative splicing transcriptome” [53].

#### 1.2.2. NMD Factors are Essential in Embryonic Development

The role of the NMD pathway during embryogenesis and during neuronal development has recently been reviewed [54]. Lou et al. studied a human embryonic stem cell line (ESC) and found that NMD factors are highly expressed in pluripotent stem cells, showing that NMD aids pluripotency in human ESCs [55]. In mammalian cells, disruption in UPF1 [56], UPF2 [57] and SMG6 [58] results in embryonic lethality in mice. Additionally, it has been proven that SMG1 is crucial for embryogenesis and that it influences the regulation of target genes via alternative splicing coupled to NMD. The study demonstrated a vital SMG1 function in early mouse development and linked the loss of this NMD component to the widespread changes in the mammalian transcriptome [59].

It has been discovered that disruptions in NMD components during development can result in male infertility. Downregulation of *UPF2* gene at the early stages of spermatogenesis leads to loss of NMD and disappearance of the vast majority of spermatogenic cells. Interestingly, when UPF2 gets disrupted during postmeiotic stages, there is a decrease in NMD activated by long 3΄ UTR, but EJC-associated NMD is not disrupted [60].

#### 1.2.3. Cellular Responses to Stress through NMD Regulation

The NMD pathway takes part in many crucial physiological processes, such as the response to stress, regulation of the immune system and viral replication. In the context of stress, NMD is incorporated among several strategies that are designed to counter the stress and restore homeostasis, or alternatively to divert cell-fate towards apoptosis. Stress mitigation can involve the repression of NMD as seen for stresses including nutrient deprivation, hypoxia or infection [15,53]. Inhibition of NMD is partially mediated by phosphorylation of the translation initiation factor eIF2, which stops the translation process and is a common step in several stress response schemes [61]. Downregulation of NMD pathway activity allows for the accumulation of NMD-targeted transcripts, including stress-response-factors and this increases cell survival [61].

NMD also acts as a regulator of the unfolded protein response (UPR), which is activated by endoplasmic reticulum (ER) stress. ER-stress is an imbalance between protein folding demand and the folding capacity of ER that results in the increased amount of unfolded proteins. While a physiologically beneficial pathway, the UPR requires tight regulation to avoid deleterious consequences. A persistent activation of the UPR is a cause of a wide variety of human diseases, therefore the unfolded protein response pathway must undergo a strict regulation. In a physiological state, NMD protects cells from the overactivation of UPR. Under stress conditions, NMD inhibition triggers UPR response through the upregulation of UPR sensors such as IRE1. However, as a consequence of inhibition, an accumulation of truncated misfolded proteins in the ER, leads to a cellular stress signal. NMD re-activation then helps end the stress response and leads to homeostasis [53]. 

#### 1.2.4. NMD as a Regulator of the Immune Response and Viral Replication

The NMD pathway also plays an important role in the regulation of the immune system [62]. Signalling molecules (cytokines) that modulate the inflammation in response to infection are upregulated. The binding of cytokines to their receptors at the cell surface initiates an immune response cascade. It has been observed that UPF1, along with the RNA-binding protein, Regnase-1, degrades cytokine mRNAs at the first stages of the inflammation response. Regnase-1 binds to mRNAs and acts with UPF1 to downregulate these transcripts. On the other hand, RNA-binding protein Roquin functions in UPF1-independent manner to regulate the late phase of inflammation [62].

It has been shown that the NMD pathway controls the stability of transcripts encoding cytokine receptors; thus, influencing the inflammation response. mRNA of human CCR5 cytokine receptor harbors a programmed −1 ribosomal frameshift (−1PRF) signal, which directs the translating ribosome to a PTC, leading to mRNA downregulation [63].

Along with its role in immune response regulation, NMD also serves as a natural barrier to virus replication. Research conducted by Balistreri et al. show that downregulation of *UPF1*, *SMG5* and *SMG7* increases the level of viral protein and leads to higher viral infection. This concludes that NMD function is important for the antiviral response, providing a first line of cell defence before virus sensing and induction of other effectors [52,64]. 

Viruses have also evolved mechanisms to escape NMD-mediated degradation. One way to escape the decay pathway is through integrating a stability element in 3′ UTR that may prevent UPF1 function [65]. On the other hand, the human T-cell lymphotropic virus type 1 (HTLV-1) virus uses Tax and Rex proteins to inhibit NMD by interaction with UPF1. Tax induces the accumulation of phosphorylated UPF1 in P-bodies which leads to enhanced stability of HTLV-1 mRNAs [66]. Waga et al. demonstrated that mRNA of coronaviruses (CoV) show multiple features that subject them to the NMD pathway, such as multiple open-reading frames (ORFs) with internal STOP codons that make up a long 3′ UTR. This led to the conclusion that CoVs evolved a strategy to inhibit the NMD pathway. The study identified that nucleocapsid protein inhibits NMD protecting viral RNAs from decay and leading to the efficient replication of the coronavirus. The process of NMD inhibition has been shown to promote accumulation of viral transcripts at the early stage of infection. Considering the role that NMD plays in regulation of stress and immune responses, inhibition of host NMD activity by nucleocapsid protein contributes to the pathogenicity of CoVs [67].

The mechanism of NMD restriction of viral replication by destabilizing viral transcripts containing internal stop codons or long 3′ UTRs has also been observed in plants. Similarly, plant viruses have also evolved ways to escape NMD, or modify host endogenous NMD activity [66]. This proves that the host NMD response reducing viral infection is an evolutionary conserved process, as well as the modulation of the NMD pathway counteracted by viruses.

A broad bioinformatic analysis on the RNASeq data from human cell lines with knockdowns of key NMD components *UPF1*, *SMG6* or *SMG7* demonstrated that the endoneucleolytic (*SMG6*-mediated) and exonucleolytic (*SMG 5/7* mediated) decay routes are redundant, degrading mRNAs with introns in 3′ UTR regions [43]. It has been observed that NMD targets with 5′ upstream open reading frames (uORFs) and long 3′ UTRs tend to be categorized as transcription factors, stress response genes and oncogenes [31,68].

A better understanding of NMD regulation of the immune response might lead to new therapeutic strategies to fight immune-related diseases including autoimmunity and immune-evasion by cancer. For example, upregulating the NMD pathway can help to bring back the immune homeostasis during chronic inflammation [62].

### 1.3. Nonsense-Mediated mRNA Decay and Genetic Disease

Of the 30 megabases that constitute human exonic sequences, 12% of single nucleotide mutations could generate transcripts with PTCs [69]. It is no surprise then that several such germline-variants in the population are implicated in diseases, including cystic fibrosis, ß-thalassemia and Duchenne muscular dystrophy [70]. In human genetic diseases, NMD can play two roles through the degradation of the PTC containing transcript. Beneficially, and in the context of feedback loops that increase dependence on the healthy transcript, NMD can limit the effects of a dominant negative mutation. However, if the PTC would have resulted in a partially functional but truncated protein, the destruction of this transcript by NMD could increase the severity of the disease [71].

ß-thalassemia is a disease caused by a defective ß-globin gene, often due to nonsense mutations in the gene. Heterozygotes with PTCs in the first or second exon are often asymptomatic owing to NMD downregulation of the mutated gene in red blood cells. NMD buffers the mutation with sufficient amounts of ß-globin by favouring the normal allele. On the other hand, PTCs in the final exon of ß-globin seem to escape the NMD pathway leading to high levels of the truncated transcript. The protein surveillance system in red blood cells fails to remove these truncated ß-chains, causing a clinical phenotype in the heterozygote called thalassemia intermedia [72,73]

In mammalian cells, *UPF3b* expression changes during brain development, and mutations in *UPF3b* and impaired NMD function inhibit proper neurite outgrowth [74,75]. It has been observed that patients carrying *UPF3b* mutations display facial dysmorphism, neurological abnormalities including schizophrenia or autism. Interestingly, the degree of mental retardation among those patients, depends on the amount of UPF3a protein produced in response to UPF3b deficiency [74].

In another example, overexpression of the double homeobox transcription factor DUX4, observed muscular dystrophy, facioscapulohumeral muscular dystrophy (FSHD), leads to activation of UPF1 proteolytic degradation and, therefore, NMD inhibition. DUX4 mRNA is also an NMD target, which means that through a double-negative feedback loop inhibition of NMD by DUX4 protein results in stable DUX4 transcript in FSHD muscle cells [76].

Stop-codon readthrough has been the main therapeutic approach pursued to treat NMD-implicated diseases. This strategy depends on small-molecule read-through agents [77] that induce the translation machinery to ignore the PTC and instead recode it as an amino acid. The resulting full-length protein is potentially functional, ameliorating the disease. Aminoglycosides were used to cause read-through of termination codons by the misincorporation of amino acids corresponding to near-cognate transfer RNA (tRNA) [78]. Gentamicin, another readthrough agent, has been utilized in clinical trials for cystic fibrosis and Duchenne muscular dystrophy. These trials showed the efficacy of this readthrough agent in restoring protein function. Unfortunately, the high doses of gentamicin required to achieve clinical utility in treatment may have adverse effects [79,80]. Ataluren has been pitched as an alternative with reported selectively in promoting PTC read-through over normal stop codons. However, the molecular mechanism of action has been challenged and its future use in clinics will largely depend on the patient outcomes of currently ongoing clinical trials [81].

### 1.4. A Dual Role for NMD in Cancer

There is growing evidence that the magnitude of NMD components are often inhibited in cancer cells. The role of NMD pathway in tumours is complex. NMD can both protect against disease and aggravate the disease phenotype, depending on the nature of the mutation triggering NMD and the type of the disease itself. This dual function of NMD has been documented by many studies focusing on NMD’s effects in human inherited genetic diseases [53]. Tumours exploit NMD to downregulate tumour-suppressors expression by selecting for mutations causing destruction of their transcripts and on the other hand, cancer cells adjust NMD activity to adapt to the tumour microenvironment. Understanding how certain tumours use the NMD pathway for their benefit may help in the development of new therapeutic interventions.

#### 1.4.1. NMD as a Protective Agent in Cancer

In cancer, NMD can again act to buffer and hide aberrant mutations that are carried forward as the disease progresses. This can be beneficial, for example if the transcript encodes for tumour promoting dominant negative truncated forms of tumour-suppressor proteins. Through degradation of those PTC-carrying transcripts, the NMD pathway protects heterozygous germline carriers of these cancer mutants from developing cancer [82]. This phenomenon has been well documented for dominant negative heterozygous mutants of the tumour suppressor gene *BRCA1*, where germline mutations in this gene lead to familial cases of ovarian and breast cancer [83]. If expressed, the truncated BRCA1 has been shown to cause chemoresistance, decreased susceptibility to apoptosis, decrease in vivo tumour growth suppression, as well as inhibition of “protective” estrogen receptor transcriptional activity, suggesting that truncated BRCA1 proteins function by inhibiting the activity of wild-type BRCA1 [84]. 

#### 1.4.2. NMD Implied in Cancer Aggressiveness and Progression

In contrast, targeted degradation of a fully- or partially- functional tumour suppressor proteins by the NMD pathway has a potential to increase the severity of cancer. As an example, the adhesion protein E-cadherin retains partial functionality when truncated at the C-terminus [85]. In the case of hereditary diffuse gastric cancer (HDGC), germline mutations in E-cadherin (*CDH1*) gene can often lead to PTC generation. Activation of NMD can reduce the level of the “protective” protein [86]. A bias is observed for patients harbouring *CDH1* mutations at the extreme 3′ end of the gene that are able to escape from NMD. These patients have a reduced risk of developing HDGC compared to people harbouring NMD-activating *CDH1* mutations [86,87].

Aberrations directly affecting the NMD machinery or the abundances of NMD components also occur in cancer, indicating that disruption of normal NMD functionality can sometimes be tolerated or promotes the disease. For example, somatic mutations in the *UPF1* gene in pancreatic adenosquamous carcinoma (ASC) tumours have been described [88]. These somatic point mutations were clustered in two regions of the *UPF1* gene. Many seem to trigger alternative splicing of the *UPF1* pre-mRNA, leading to the expression of truncated UPF1 proteins [81]. Similarly, UPF1 expression is shown to be lower in lung adenocarcinoma (ADC) when compared to normal tissue. This characteristic leads to the decrease of NMD activity, which can lead to the upregulation of genes typically under NMD control. For example, transforming growth factor beta (TGF-β) signalling components, which are vital to the epithelial-to-mesenchymal transition (EMT). During EMT, epithelial cells lose their cell polarity and cell-cell adhesion [89].

It has been discovered that SMG1 expression negatively correlates with HPV (Human Papillomavirus) status in cancer cell lines and tumours. HPV is linked with a subset of head and neck squamous cell carcinomas (HNSCCs), where patients with HPV-positive tumours show a better prognosis than HPV-negative HNSCCs, which may be explained by increased sensitivity of the HPV-positive HNSCCs to ionizing radiation (IR). Gubanova et al. showed that low SMG1 level results in elevated sensitivity to ionizing radiation due to increased induction of apoptosis in HPV-positive HNSCCs [90]. Depletion of SMG-1 in HPV-negative HNSCC cells resulted in increased radiation sensitivity, while SMG-1 overexpression protected HPV-positive tumour cells from irradiation.

Tumour evolution is also impacted by NMD, which can buffer lethal passenger mutations arising as the tumour explores sequence space. So, as tumours progress, they may become addicted to the NMD pathway through its capacity to conceal deleterious events. As sequence space is explored, tumour-suppressor genes can exhibit a high number of nonsense mutations triggering NMD and, therefore, lower the level of beneficial tumour suppressor proteins [91]. In contrast, oncogenes can harbour missense mutations that do not elicit NMD. In these cases, NMD-escape allows for elevated oncoprotein expression [91].

#### 1.4.3. Role of NMD in the Tumour Microenvironment

NMD modulation has been observed in the tumour microenvironment to influence the fate of the tumour. Wang et al. identified ~750 mRNAs that were significantly upregulated in osteosarcoma cells in response to NMD inhibition. The scientists tested RNA-mediated depletion of UPF1 or UPF2, and exposed cells to hypoxia, or incubated with the ER stress inducer tunicamycin [60]. The data showed that among the upregulated transcripts, proteins involved in tumour-promoting pathways were overrepresented. Many of these NMD substrates have been shown to be upregulated in in vivo tumour models, as well as in Burkitt lymphoma, melanoma, breast and prostate cancers.

In prostate cancer cells, overexpression of UPF1 is shown to make cells no longer sensitive to NMD inhibition by cellular stress, do not grow as three-dimensional spheres or as xenografts in nude mice; however, their growth in standard tissue culture conditions is unaffected. The study suggests that NMD inhibition by the tumour microenvironment is an important mechanism of gene regulation crucial for tumourigenesis and for dictating the outcome of malignancy [60].

### 1.5. NMD Inhibition in Cancer Therapy

The development of inhibitors of the various components of the NMD pathway is critical for gaining mechanistic insights into NMD function and provides new avenues for therapeutic intervention. Lindeboom et al. analysing a pan-cancer cohort, found that the burden of frameshift mutations that do not trigger NMD correlates with increased tumour immune reactivity, which suggests that these tumours may also respond better to immunotherapy [92]. Together with the enhanced immune reactivity of tumours with mutations in the NMD pathway, this suggests that inhibiting NMD may serve as an effective strategy to potentiate the efficacy of checkpoint inhibitors. By preventing the destruction of PTC-containing transcripts, reduced NMD activity may enhance the expression of tumour antigens that can be recognized by the immune system as “foreign”-neoantigens [93].

Another study has identified potential benefits within a subgroup of colorectal cancers (CRC) characterized by high levels of mRNAs with a PTC, a consequence of microsatellite instability (MSI) in this disease. Inhibition of NMD in vivo using amlexanox reduced cell proliferation and had an antitumour effect on MSI tumour xenografts. Interestingly, no major effect was seen in the microsatellite stable colorectal cancer cell lines [94]. 

Central tolerance is the process of eliminating any developing T or B lymphocytes that are reactive to self. It has been proposed that new peptides produced as a result of aberrant splicing or NMD-dependent autoregulated alternative splicing elicit T-cell responses not subject to host central tolerance in the thymus, because of the lack of expression in healthy tissues. Therefore, some of the transcripts, when NMD is inhibited in tumour cells, are able to induce an immune response and inhibit tumour growth. Furthermore, it has been observed that frameshift mutations in tumour cells that exhibit DNA mismatch repair produce PTC-containing mRNAs negatively controlled by NMD [95]. Therefore, NMD inhibition could then stimulate the production of tumour-specific antigens.

#### Development of NMD Inhibitors

The search for NMD inhibitors has yielded a few good candidates with varying effects. A small molecule termed NMDI 1, stabilizes the hyperphosphorylated isoforms of the UPF1 protein, and reduces the interactions between UPF1-SMG5 proteins [94,95,96]. Moreover, compounds that can compromise the SMG7-UPF1 interactions have also been identified, and combining these compounds with a PTC read-through drug enhances the restoration of full-length p53 [95,97]. Making direct interactions with the EJC component; eIF4AIII, the pateamine A (PatA) (natural product) inhibits NMD [96,98]. Curcumin is a dietary compound that inhibits NMD by downregulating its expression at transcriptional level [97,99]. Pyrimidine derivatives have also been identified as inhibitors against the SMG1 protein [100]. Upon blocking NMD, the inhibitors (wortmannin and caffeine) were found to increase the expression of the mutant collagen VI α2 subunit [99,101]. 

Cardiac glycosides (e.g., digoxin, ouabain) increase cytoplasmic calcium, which represses NMD pathway [100,102]. Moreover, a screen in HeLa cells stably expressing an NMD reporter that used a library of clinically licensed compounds identified 5-azacytidine (an analog of the naturally occurring nucleoside cytidine), which has been previously approved for the treatment of myelodysplastic syndrome and myeloid leukaemia [103]. The inhibitory effects of this compound depends on the induction of MYC expression (it is shown to inhibit NMD) [104].

Translation is necessary for NMD [107]; thus, it is suggested that inhibitors of translation may also be effective inhibitors of NMD [104]. Moreover, Martin et al. have recently demonstrated that NMD inhibition can be achieved via other mechanisms [16,93] and also determined that 80% depletion of UPF1 can suppress NMD activity not influencing the proliferation or survival of cells [55,93]. Therefore, inhibitors that can modulate NMD activity offer critical tools for understanding the mechanism and physiological functions of the NMD pathway [75,108]. Targeting protein-protein interaction binding sites is crucial in drug discovery, since many physiological and pathological cellular processes depend on protein-protein interactions that can be influenced by compounds or peptide-like molecules [16]. Although, previous studies have identified NMD inhibitors with different mechanisms of action (e.g., NMDI-1 disrupts UPF1-SMG5 interactions), there are several interactions and active-sites left to exploit and study. Three-dimensional protein structures can reveal the precise interactions defining the protein-protein interface for *in silico* drug design, which can be targeted for drug discovery [109,110,111]. These protein-protein structures available in the Protein Data Bank (http://www.rcsb.org/pdb) [111] for NMD pathway are (Figure 2 and Table 1): UPF1-UPF2 [34], UPF2-UPF3b [36], SMG5-SMG7 [103,105,112], SMG8-SMG9 [104,106], SMG1–SMG8–SMG9 [107] and the EJC (Mago-Y14-eIF4AIII-Barentsz-UPF3b) [108]. Figure 2, illustrates different protein-protein or protein-RNA interactions from the NMD pathways that could represent a base or the platform to design inhibitors or peptide-like molecules.

## 2. Conclusions

Nonsense-mediated mRNA decay is a critical RNA quality control and plays a vital role in the recognition of PTCs in transcripts, as well as in the regular homeostasis of the transcriptome. NMD has been implicated during the development, DNA damage response, cell cycle regulation and in immune system function. Because of its involvement in maintaining the genome stability, disruptions in this strictly regulated surveillance pathway lead to pathologies including neurological disorders, immune diseases and cancers. The role of NMD in cancer development is complex. In some cases, NMD acts to support tumour growth by downregulation of key tumour suppressor genes. In other cases, tumours adjust NMD activity to adapt to their microenvironment [2].

Cancer-targeted NMD inhibition might represent a new strategy to promote protective anti-tumour immunity. Antigens expressed on tumour cells may be less immunogenic in an effort to avoid the immune-system. Instead of stimulating immune responses to these less effective antigens, the goal of novel tumour vaccination protocols is to generate new antigenic determinants through inhibition of the NMD. In order to design the most effective NMD inhibitor, the structural aspects of this pathway must be studied, such as different protein-protein or protein-RNA interactions. Understanding the mechanism of nonsense-mediated mRNA decay might lead to new ways to use surveillance in cancer therapy or other PTC- associated genetic diseases.

## Figures and Tables

**Table 1:** The crystal structures available for the protein-protein binding interface available for different NMD components, from the Protein Data Bank database (http://www.rcsb.org/pdb) [111]. UPF: up-frameshift; SMG: suppressor of morphogenetic effect on genitalia.

Protein Interacting Partners	PDB ID.	Resolution	Method	References
UPF1-UPF2	2wjv	2.85 Å	X-Ray diffraction	[34]
UPF2-UPF3b	1uw4	1.95 Å	X-Ray diffraction	[36]
SMG5-SMG7	3zhe	3 Å	X-Ray diffraction	[112]
SMG8-SMG9	5nkk	2.64 Å	X-Ray diffraction	[106]
SMG1–SMG8–SMG9	6syt	3.45 Å	Electron microscopy	[107]
Mago-Y14-eIF4AIII-Barentsz-UPF3b	2xb2	3.4 Å	X-Ray diffraction	[108]
